# Rheumatoid Arthritis and Anti-Thyroid Antibodies

**DOI:** 10.1080/08916930802428114

**Published:** 2009-01-20

**Authors:** Irfan Yavasoglu, Taskin Senturk, Adil Coskun, Zahit Bolaman

**Affiliations:** 1 Department of Internal Medicine, Medical School of Adnan Menderes University, Aydin, Turkey; 2 Department of Rheumatology, Medical School of Adnan Menderes University, Aydin, Turkey

Irfan Yavaşoğlu 4.8.2008

Editor:

The article entitled “Anti-thyroid antibodies and thyroid dysfunction in rheumatoid arthritis (RA): prevalence and clinical value” written by Atzeni et al. [1] and published in one of the recent issues of your journal was quite interesting. Here, we would like to emphasize some points.

RA is a chronic inflammatory autoimmune disorder that affects approximately 0.5–1% xof the population. According to general opinion systemic autoimmune disorders rarely exists with organ specific autoimmune disorders. Our study included 82 patients (67 females and 15 males) diagnosed as RA according to criteria of ARA as well as 47 healthy control subjects (31 females and 16 males). All participants' free thyroxine (fT4), free triiodothyronine (fT3), thyroid-stimulating hormone (TSH), thyroglobulin antibody (anti-Tg) and anti-peroxidase (anti-TPO) antibody titers were measured. Results of the RA patients were compared with the controls (Table I). The anti-TPO positivity were 15.9% (13 patients) in patients with RA and 2.6% in control group. The anti-Tg positivity were 12.3% (10 patients) in patients with RA and 1.8% in control group [2]. Other study (like Turkish population), the overall frequencies of thyroid antibodies were 11% in primary Sjögren's syndrome, 7% in RA, 17% in secondary Sjögren's syndrome, 8% in healthy controls, and 94% in autoimmune thyroiditis.

**Table I:** The thyroid test findings for RA and controls.

	RA (*n* = 82)	Control (*n* = 47)	*p*-value
fT3 (1.82–4.62 pg/ml)⋆	2.72 ± 0.79	2.56 ± 0.71	>0.05
fT4(0.932–1.71 ng/dl)⋆	1.21 ± 0.29	1.19 ± 0.28	>0.05
TSH (0.270–4.2 IU/ml)⋆	3.46 ± 1.92	3.06 ± 1.52	>0.05
Anti-TPO (>40 IU/ml)⋆	85.69 ± 106.83	43.16 ± 132.23	0.008
Anti-Tg (>40 IU/ml)⋆	58.2 ± 83.9	24.19 ± 36.74	0.0004

⋆ Normal value.

There was no difference for the frequency of the thyroid antibodies among the groups if patients with autoimmune thyroiditis were excluded [3]. Control group is important. Racial difference (even local) may exist between RA and anti-thyroid antibodies. In our area there is endemic goiter. Although rheumatoid factor (RF) is an autoantibody, in the current study we found no correlation between RF titer and duration of RA thyroid autoantibodies in RA patients (*p* > 0.05). We suggest that anti-thyroid autoantibodies are independent from the duration of RA and RF as there was not an apparent finding specific for a thyroid disorder in patients with RA in our study [2]. In conclusion, the anti-thyroid antibody positivity identified here might be due to a pathologic auto-immune response rather than demonstrating a thyroid disease. Thyroid function and anti-thyroid antibodies tests should be performed as part of the biochemical and immunological profile in RA patients.
